# Case Report: Abnormalities of sperm motility and morphology in a patient with Leber hereditary optic neuropathy: Improvement after idebenone therapy

**DOI:** 10.3389/fneur.2022.946559

**Published:** 2023-01-04

**Authors:** Christophe Orssaud, Virginie Barraud Lange, Jean Philippe Wolf, Nathalie LeFoll, Jean Claude Soufir

**Affiliations:** ^1^Functional Unit of Ophthalmology, Ophtara Rare disease Center, Sensgène Filière, ERN EYE, European Hospital Georges Pompidou, University Hospital Paris Centre, Assistance Publique de Hôpitaux de Paris, Paris, France; ^2^Team Genomic Epigenetic and Physiopathology of Reproduction, Department of Genetic, Development and Cancer, Cochin Institute, Inserm U1016, Paris, France; ^3^Laboratory of Histology Embryology Biology of Reproduction, Sorbonne Paris Cité, Faculty of Medicine, University Hospital Paris Centre, Assistance Publique-Hôpitaux de Paris, Paris, France; ^4^Biologie de la Reproduction, University Hospital Paris Centre, Assistance Publique Hôpitaux de Paris, Paris, France

**Keywords:** Leber hereditary optic neuropathy, spermatogenesis, sperm motility, idebenone, mtDNA mutation

## Abstract

**Case:**

We report the sperm characteristics of a male patient who developed, when he was 18 years old, a Leber hereditary optic neuropathy, a hereditary optic neuropathy due to mtDNA mutation as well as variants in the nuclear DNA. At the age of 30 years-old, he complained of infertility lasting for 2 years. Semen analyses showed low motility spermatozoa and a high percentage of morphological or ultrastructural abnormalities. Levels of epididymal markers were strongly atypical. Idebenone was prescribed as treatment of his Leber hereditary optic neuropathy in order to improve his visual acuity. After 5 months of this treatment, motility of spermatozoa increased, and their vitality improved. A natural conception occurred.

**Outcome:**

This case is the first description of an anomaly of spermatozoas and of the epididymis epithelium in a patient with Leber hereditary optic neuropathy. It draws attention to sperm pathologies in patients with mitochondrial disorders. The role of the mtDNA mutations must be suspected since it plays an important role in the development and motility of spermatozoa. In addition, idebenone can by-pass the complex I and transfer electrons to complex III. It has been suspected to have a favorable effect on spermatogenesis.

**Conclusion:**

This case confirms the possibility of sperm dysfunction in Leber hereditary optic neuropathy and the interest of idebenone as a treatment for infertility due to mtDNA mutations in human.

## 1. Introduction

Leber Hereditary optic neuropathy (LHON) is an optic nerve dysfunction transmitted in a non-mendelian pattern as well as in an autosomal recessive mode (1). Three mitochondrial mutations in mitochondrial DNA (mtDNA): 3,460, 11,778, and 14,484 account for more than 90% of all LHON cases (2). In addition, recessively inherited defects in the DNAJC30 gene, that affect complex I function, or in the MCAT gene have been reported (3). Sequential bilateral visual failure is due to focal degeneration of retinal ganglion cells leading to optic atrophy. LHON begins in more than 70% of the case between 18 and 35 years of age, predominantly in males.

LHON mutations lead to abnormalities in the complex I of the mitochondrial respiratory chain. Altered function of this complex causes some decrease in ATP synthesis, but most importantly an increase in free radical production and oxidative damage. The predominant manifestation of LHON is an optic atrophy. Further evidence was provided that LHON can occasionally affects (4). Different treatments are proposed in LHON: idebenone which is an approved medication in Europe and an intravitreal gene therapy with a recombinant adeno-associated virus vector serotype 2 (rAAV2) containing a codon-modified complementary DNA (cDNA) encoding the human wild-type mitochondrial ND4 protein (5, 6).

The role of mitochondria in reproduction was suspected for a long time (7). Recently, Boguenet confirmed their important function, especially in gamete quality (8). However, such dysfunction was not reported previously in male LHON patients. In addition, it was observed that idebenone was able to promote the vitality of spermatozoa in animals (9–11). But, such treatment was never used before to treat male infertility due to mtDNA mutation in human.

We report here sperm characteristics consecutive of testis and epididymal disorders in a male LHON as well as the course of the disease after administration of idebenone, a synthetic analog of coenzyme Q (12).

## 2. Case report

2.1. A 30-year old man who developed LHON at the age of 18 years related to a mutation 11,778, consulted for infertility lasting 2 years. There was no other significant family (one brother and two sisters) or personal medical history, including no history of infertility in his family. The patient was a non-smoker.

Three semen analyses were performed before ibedenone treatment at intervals of 3–6 months. The main abnormalities were low motility and a high percentage of atypical forms (97%) of spermatozoa. Morphological abnormalities were most prominent in the flagella (absent, short and coiled) and in the middle pieces (cytoplasmic droplet) (Table 1). Moreover, ultrastructural study of spermatozoa revealed abnormal fibrous sheath in 55% of the flagella without abnormalities of microtubules doublets and dynein arms. Seminal biochemistry showed normal values of seminal vesicles and prostate secretions (especially citrate synthetized in the mitochondria of the prostatic glandular tissue). In contrast, levels of epididymal markers were strongly discordant: normal for alpha 1–4 glucosidase but greatly reduced free L-carnitine (Table 1).

**Table 1:** Results of semen analyses before any treatment, after embolization of varicocele, and after idebenone treatment.

	**Normal**	Before treatment^*^	**After embolization**	**After idebenone treatment**		
				**3 Mo** ^*^	**4 Mo** ^**^	**32 Mo**
Volume mL	1.5–7.6 mL	4.9	6	3.9	4	4
Sperm concentration, 10^6^/mL	15–259	9	9	11	14	8.4
Total sperm number, × 10^6^	39–928	44	54	44	44	34
**Motility, %**						
Total motile	40–81%	10	22	37	38	30
Progressive	32–75%	5	15	23	23	15
Nonprogressive		5	7	14	15	15
Vitality %	>75%	60	52	72	74	80
Atypical spermatozoa, %		0	8	7	7	2
**Tail defects %**						
Absent		9	10	6	7	10
Short		24	8	15	11	15
Irregular width		11	5	11	11	14
Coiled		34	43	19	31	23
Middle piece defect %						
Cytoplasmic droplet		29	25	15	14	17
**Epipidymal seminal markers**						
Free L-carnitine, nmol		331	364		193	
α-1-4-glucosidase, mU		77	56		65	

* Mean value from 2 semen analyses.

** Mean value from 3 semen analyses.

2.2. Karyotype was normal. Scrotal utrasonography revealed bilateral varicocele predominant of the left side. Testes were normal, epididymides were flat and featureless. Varicocele was treated by embolization. Six months after this intervention, there was a very limited improvement in the sperm characteristics.

2.3. Ten months later, this patient consulted to be informed of new therapy for LHON. A treatment consisting of idebenone (60 mg/day) in combination with vitamin B2 (30 mg/day) and vitamin C (1 g/day) was introduced according to the recommendations of Mashima (13).

2.4. Five semen analysis were performed between the third and fifth month of treatment. Motility increased by 2–2.5 for total motile and by 3 to 4 for progressive motile and vitality normalized (Table 1). Atypical forms were reduced by 50–65%, the greatest improvement occurring after 5 months of treatment. In contrast, the percentage of atypical flagella was not reduced. Abnormal epididymal biochemistry (carnitine deficiency) was unchanged under idebenone treatment.

After 30 months of treatment, natural conception occurred. Semen analysis realized at this same time confirmed the improvement of sperm parameters.

## 3. Discussion

3.1. To our best knowledge, it is the first description of an infertility in a LHON patient due to sperm abnormalities. Occurrence of dysfunction of the testis is coherent to mutation of the mtDNA as pointed out by Boguenet (8). A perturbation of the mitochondrial function alters the germ line survival and the epididymis epithelium (8). Different mechanisms could be mentioned.

3.2. Oxidative stress is known to be a factor leading to infertility (14). However, existence of an oxidative stress in the pathophysiology of LHON was proved by Rovcanin in a large series of patients (15). Efficacy of drugs reducing ROS is another argument in favor of the role of oxidative stress during this pathology (16).

Defects in the elimination of residual cytoplasm, arrest of flagellum morphogenesis and fibrous sheath dysplasia are generally due to genetic disturbances (17, 18). Variability in mtDNA content or mtDNA deletions are associated with motility disorders and midpiece abnormalities explained by a deficit of respiration during spermatogenesis (7, 19). Impairment of motility and flagellar abnormalities (coiled) could be due to a hostile endogenous milieu in the epididymis (20). In our case, epididymal function was disrupted. Carnitine uptake, related to the acquisition of motility by spermatozoa, was much reduced or absent. This deficiency could partly explain impairment of motility in sperm with normal flagella. However, we did not observe an improvement of carnitine level after idebenone treatment. This could explain that the percentage of sperm mobility increased but remained lower than normal.

3.3. Coenzyme Q10 (CoQ10) or its active derivative, ubiquinol had been indicated to treat male infertility. It was prescribed alone or combined with other compounds considered as antioxidants (L-carnitine, vitamin C, vitamin E, zinc, vitamin B9, selenium, vitamin B12) (21). Improvement in sperm motility usually occurred after 6 months in patients with asthenozoospermia (22).

CoQ10 is synthesized by all cells in the body. It is a quinone associated with a chain of 10 isoprene units. It is therefore insoluble in aqueous solutions. Lipophilic, it is inserted into cell membranes and more particularly into the inner membrane of the mitochondria where it transports the electrons of complexes I and II to the complex III. CoQ10 is considered a powerful physiological antioxidant protecting cell membranes against free radical aggression.

3.4. Idebenone is a synthetic quinone. It has a short isoprene chain with a terminal hydroxyl function which polarizes the molecule. This structure makes it soluble in an aqueous medium. Although they are part of the same family, benzoquinones, CoQ10 and ibedenone act differently and are not substitutable. While the absorption, pharmacokinetics, distribution and metabolism of CoQ10 remain problematic, idebenone present a blood peak 3 h after its absorption. Its half-life is between 10 and 13 h with a linear pharmacokinetics. Its human tolerance is excellent for doses ranging from 1,000 to 2,000 mg.

The action of idebenone depends on its reduction by NQO1, a flavoprotein essentially present in the cytoplasm (23). Therefore, due to its solubility, the action of idebenone is not limited to membranes: it can transfer electron equivalents from the cytoplasm to the mitochondria. As CoQ 10, idebenone cannot transfer electrons from complex I to III (NADH dehydrogenase). Due to its solubility, it can transfer electrons from the cytoplasm directly to complex III. Thus, idebenone can then by-pass the complex I. This action is advantageous when dysfunctions occur at the level on complex I, as in LHON. The peroxidation of the lipids of the mitochondrial membranes disrupts the reduction of Co Q10, while it does not interfere with the activation of the idebenone since its localization is cytoplasmic.

3.5. The antioxidant effect of idebenone was demonstrated *in vivo*, unlike that of CoQ10. However, this action requires concentrations that can vary by a factor of 100, from one cell type to another. In addition, NQO1 is expressed very unequally in the different tissues. However, its presence is well documented in the male genitalia. In humans, NRf2 which regulate NQO1 is expressed in the vas deferens, testis and epididymis which make these organs a good target for idebenone (24).

Several recent publications tend to prove that this molecule could have a beneficial effect on the conservation of cryopreserved semen to promote the vitality of spermatozoa due to its action on oxidative stress (10, 11). In addition, idebenone reduced sperm ROS concentrations associated with advanced paternal age and improved fertilization rates, embryo quality and implantation rates after *vitro* fertilization in mice (9).

3.6. Due to the lack of clear improvement in the spermogram 6 months after the treatment of the varicocele, it could be considered that this malformation played only a modest role in this infertility. Usually, most of the effect of its treatment occurred usually within the first 3 months (25). There are many data showing that varicocele induced a poor sperm quality and infertility due to oxidative stress (26). Thus, it is likely that the varicocele increased the oxidative stress induced by the mtDNA mutation. Infertility in this patient with LHON would therefore be due to the conjunction of two abnormalities potentiating each other with nevertheless a predominance of the role of the LHON and the mutation of the mtDNA.

3.7. The dose of idebenone prescribed to our patient was lower than what is actually recommended (13). After oral administration, <1% of administrated idebenone reaches the systemic circulation (9). Therefore, there is uncertainty regarding the tissue concentration obtained In addition, we cannot exclude that intake of vitamin C participate to the improvement of the fertility in our patient. However, vitamin C can act as free radical scavengers and thus reduce oxidative stress. But it seems principally to protect sperm cells against mtDNA fragmentation (27, 28). This property is less important in our case with mtDNA mutation. On the other hand, vitamin B2 does not seem to be involved in the protection against male infertility.

But this case illustrates for the first time the effect of this molecule on fertility itself *in vivo* in human. It can therefore be considered as a treatment route for sterility or infertility due to damage to the mitochondrial genome.

## Data availability statement

The original contributions presented in the study are included in the article/supplementary material, further inquiries can be directed to the corresponding author.

## Ethics statement

Written informed consent was obtained from the individual(s) for the publication of any potentially identifiable images or data included in this article.

## Author contributions

CO was the ophthalmologist of the patient and wrote the manuscript. VB and JW analyzed the sperm analysis and reviewed the manuscript. NL and JS performed the sperm analysis. JS reviewed the manuscript. All authors contributed to the article and approved the submitted version.
